# Construction of a preoperative nomogram model for predicting perineural invasion in advanced gastric cancer

**DOI:** 10.3389/fmed.2024.1344982

**Published:** 2024-06-07

**Authors:** Ruochen Cong, Ruonan Xu, Jialei Ming, Zhengqi Zhu

**Affiliations:** ^1^Department of Radiology, Nantong No. 1 People’s Hospital, Nantong, China; ^2^Department of Radiology, Nantong No. 6 People’s Hospital, Nantong, China; ^3^Department of Radiology, Nantong City Cancer Hospital, Nantong, China

**Keywords:** advanced gastric cancer, perineural invasion, predict, nomogram model, risk

## Abstract

**Objective:**

This study aimed to develop and validate a clinical and imaging-based nomogram for preoperatively predicting perineural invasion (PNI) in advanced gastric cancer.

**Methods:**

A retrospective cohort of 351 patients with advanced gastric cancer who underwent surgical resection was included. Multivariable logistic regression analysis was conducted to identify independent risk factors for PNI and to construct the nomogram. The performance of the nomogram was assessed using calibration curves, the concordance index (C-index), the area under the curve (AUC), and decision curve analysis (DCA). The disparity in disease-free survival (DFS) between the nomogram-predicted PNI-positive group and the nomogram-predicted PNI-negative group was evaluated using the Log-Rank test and Kaplan–Meier analysis.

**Results:**

Extramural vascular invasion (EMVI), Borrmann classification, tumor thickness, and the systemic inflammation response index (SIRI) emerged as independent risk factors for PNI. The nomogram model demonstrated a commendable AUC value of 0.838. Calibration curves exhibited excellent concordance, with a C-index of 0.814. DCA indicated that the model provided good clinical net benefit. The DFS of the nomogram-predicted PNI-positive group was significantly lower than that of the nomogram-predicted PNI-negative group (*p* < 0.001).

**Conclusion:**

This study successfully developed a preoperative nomogram model that not only effectively predicted PNI in gastric cancer but also facilitated postoperative risk stratification.

## 1 Introduction

Gastric cancer stands out as one of the most prevalent malignancies within the digestive system, ranking fifth globally in incidence according to the latest statistics, with approximately 738,000 annual deaths attributed to the disease (1, 2). The accurate preoperative assessment of clinical tumor staging is crucial for formulating scientifically sound treatment strategies. However, early-stage gastric cancer often manifests with subtle or no symptoms, leading to a significant number of patients being diagnosed in advanced stages (3). Compounded by various factors, preoperative assessments may fall short of expectations, resulting in concerning 5-year survival rates for gastric cancer. While radical surgical resection remains the primary treatment modality, for advanced gastric cancer, additional postoperative adjuvant chemoradiotherapy becomes necessary to control local tumor spread and reduce the risk of recurrence.

Perineural invasion (PNI) involves the infiltration of cancer cells into nerve bundles and their sheaths, representing a significant pathway for tumor invasion and metastasis (4). In patients with advanced gastric cancer, PNI is notably associated with local recurrence and poor prognosis (4, 5). Several studies (5–8) proposed that PNI served as a crucial indicator reflecting the biological behavior of gastric cancer, carrying substantial implications for enhancing tumor staging, selecting appropriate treatment plans, and assessing prognosis. Jiang et al. suggested (6) a correlation between PNI and the local spread and lymphatic metastasis of gastric cancer, advocating for its inclusion in the TNM staging system. While PNI can only be confirmed through postoperative pathology, accurately predicting the risk of PNI in preoperative gastric cancer patients can aid in identifying those who would benefit from neoadjuvant or adjuvant chemotherapy, thereby reducing recurrence and improving prognosis (5, 8, 9).

Presently, various studies formulated diverse prediction models for PNI, primarily relying on postoperative pathological outcomes. These models incorporated factors like depth of infiltration, vascular invasion, differentiation degree, and tissue type (10–13). However, such information was typically unavailable in practical clinical settings before surgery. Recent research (14–16) underscored the significant role of inflammation in tumor occurrence, development, and spread, influencing prognosis and chemotherapy effectiveness. Despite this, the correlation between inflammatory markers in peripheral blood and PNI in gastric cancer remained unclear. Moreover, with the advancement and widespread use of techniques like endoscopic ultrasound and computerized tomography (CT), clinicians can non-invasively gather valuable preoperative information, such as tumor thickness and extramural vascular invasion (EMVI), offering crucial insights into accurately determining neural invasion in gastric cancer tissue. Consequently, this study aimed to explore independent risk factors for PNI in advanced gastric cancer, constructing a preoperative risk assessment model to predict PNI. The objective was to optimize the preoperative assessment system and offer a reference point for developing more rational and accurate individualized diagnosis and treatment plans.

## 2 Materials and methods

### 2.1 Study population

**Figure 1:**
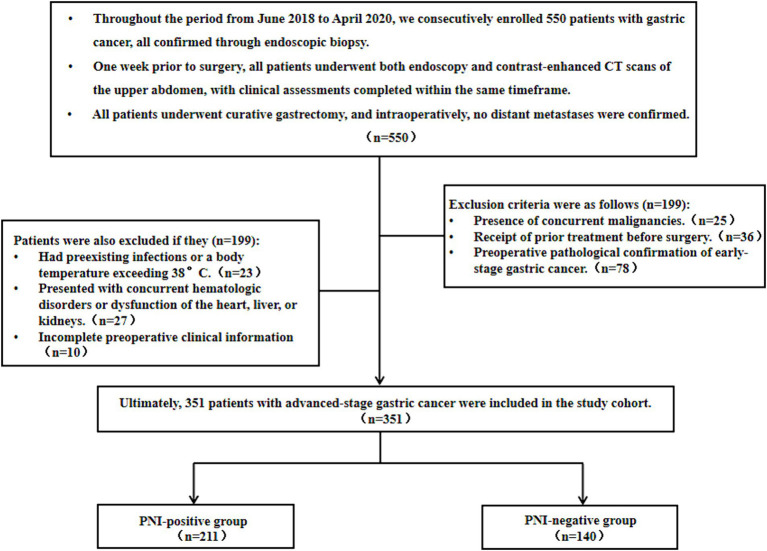
Flowchart of gastric cancer patient enrollment as well as its associated inclusion and exclusion criteria.

This retrospective cohort analysis included 351 patients with advanced gastric cancer who underwent surgical treatment at the Second Affiliated Hospital of Nantong University from June 2018 to April 2020. The cohort comprised 211 (60.11%) PNI-positive and 140 (39.89%) PNI-negative patients. The inclusion criteria for this study were as follows: (1) preoperative endoscopy and upper abdominal enhanced CT examination conducted 1 week before surgery; (2) radical gastric cancer surgery involving D2 lymph node dissection and R0 resection; (3) absence of preoperatively and intraoperatively confirmed distant metastasis. Exclusion criteria comprised: (1) concurrent or prior malignancies; (2) preoperative neoadjuvant chemoradiotherapy or immunotherapy; (3) postoperative pathology confirming early-stage gastric cancer; (4) preoperative infection or insufficient evidence of infection with a body temperature exceeding 38°C; (5) concurrent hematologic disorders or dysfunction of the liver, kidney, or heart; and (6) incomplete preoperative clinical information (Figure 1). This retrospective cohort study was conducted in accordance with the Helsinki Declaration, and all procedures were approved by the Ethics Committee of the Second Affiliated Hospital of Nantong University. Informed consent was waived.

### 2.2 Evaluation of clinical pathology and imaging features

Demographic and laboratory measurement indicators were extracted from the hospital’s electronic medical record system, encompassing details such as gender, age, tumor markers, and inflammatory markers. We computed the neutrophil-to-lymphocyte ratio (NLR), platelet-to-lymphocyte ratio (PLR), and lymphocyte-to-monocyte ratio (LMR) using the following formulas: NLR = neutrophil count/lymphocyte count (17); PLR = platelet count/lymphocyte count (18); LMR = lymphocyte count/monocyte count (19). Additionally, the systemic inflammation response index (SIRI) was determined by incorporating counts of neutrophils, monocytes, and lymphocytes in peripheral blood, utilizing the equation: SIRI = (neutrophil count × monocyte count)/lymphocyte count (20). The inflammatory markers, including NLR, PLR, LMR, and SIRI, were classified into high and low groups based on the optimal cut-off values determined by receiver operating characteristic (ROC) curves (Supplementary Table S1). Values above 5.0 ng/mL for CEA and 37.0 U/mL for CA199 were considered abnormal in our study center. Preoperative endoscopy was utilized to determine the tumor’s location, thickness, and Borrmann classification. The differentiation degree and Lauren classification of tumor tissues were elucidated based on pathology results from preoperative endoscopic biopsy. All patients were required to fast for 8–12 h, and 10 min before CT examination, 20 mg (10 mg/mL) of scopolamine was injected intramuscularly to reduce gastrointestinal peristalsis. To fully distend the stomach, patients were instructed to drink warm water (800–1,000 mL) before the CT examination. Results from preoperative CT examinations, encompassing CT T staging, enlarged lymph node on CT, and extramural vascular invasion (EMVI), were acquired using multi-planar reconstruction (MPR) on contrast-enhanced CT venous phase images.

The CT T staging criteria, as outlined by Kumano et al. (21), were as follows: ctT1 corresponds to a highly enhanced tumor that does not exceed 50% of the total gastric wall thickness, featuring a complete low-enhanced band between the tumor and the muscle layer. For ctT2, the highly enhanced tumor surpasses 50% of the total gastric wall thickness, and the low-enhanced band in the middle layer disappears. ctT3 is characterized by a highly enhanced tumor that invades the entire gastric wall, presenting a vague serosal surface or short fine strands covering less than 1/3 of the lesion area. ctT4a manifests as an irregular or nodular serosal surface with a line-like high-enhanced sign, while ctT4b involves the loss of fat interspace between the tumor and adjacent organ structures, finger-like invasion, or direct infiltration.

CT enlarged lymph node was defined as having a maximum short-axis diameter greater than 5 mm for perigastric lymph nodes (22). Positive EMVI was characterized by tumor tissue extending into the extramural vessels, resulting in vessel dilation or the presence of tubular or nodular soft tissue density within the dilated veins (23). All CT results underwent joint confirmation by two associate chief physicians with 10 years of experience in gastrointestinal imaging diagnosis. In the event of disputes, a third chief physician conducted a review of the images.

### 2.3 Diagnosis of gastric cancer PNI

Gastric cancer tissues, excised during surgery, underwent fixation in 10% neutral buffered formalin, followed by embedding in paraffin. The tissues were then sectioned into 4-μm slices and stained with hematoxylin and eosin. PNI was diagnosed under a light microscope, with the infiltration of cancer cells into any layer of nerve fibers (including the nerve outer membrane, nerve bundle sheath, and nerve inner membrane) or encircling the nerve by more than one-third considered indicative of PNI. Positive PNI was further confirmed through immunohistochemistry staining, specifically marking the nerve bundle as S-100 positive (7, 24).

### 2.4 Follow-up strategy

Patients post-surgery underwent follow-ups every 3 months within the first year and then extended to every 3–6 months until September 2023. The minimum follow-up duration for all patients was 6 months. Follow-up assessments encompassed laboratory and imaging examinations. Disease-free survival (DFS) was defined as the time interval between the tumor diagnosis and the occurrence of recurrence or metastasis, serving as the endpoint for the study.

### 2.5 Statistical analysis

Data analysis was performed using SPSS 26.0 statistical software. The normality of continuous variables was assessed using the Kolmogorov–Smirnov (K-S) test. For variables following a normal distribution, mean and standard deviations were reported, and t-tests were used for analysis. Some quantitative data in clinical indicators were converted into categorical variables, and Pearson’s chi-square test was employed for analysis. Clinical indicators showing statistically significant differences (*p* < 0.05) were included in the logistic multivariate regression model analysis [using backward regression for variable selection] to identify independent risk factors for PNI in advanced gastric cancer.

The “rms” package in the R software was employed to construct the nomogram prediction model for gastric cancer PNI. The model assigned scores to each identified risk factor, and the cumulative scores corresponded to the probability of preoperatively predicting PNI in gastric cancer. Higher scores indicated an elevated risk of PNI in patients. ROC curves were employed to assess the diagnostic efficacy of the preoperative nomogram model for gastric cancer PNI. Additionally, DeLong tests were used to compare the area under the curve (AUC) between the nomogram model and clinical indicators. Internal validation of the nomogram model was conducted using the bootstrap method with 1,000 repetitions, and the concordance index (C-index) was calculated to determine the model’s discriminatory ability. Calibration curves were generated to assess the consistency between predicted and actual results. The Hosmer-Lemeshow test was utilized to evaluate the goodness of fit of the model. The clinical utility of the model was assessed through decision curve analysis (DCA). Finally, the DFS between the nomogram-predicted PNI-positive group and the nomogram-predicted PNI-negative group was assessed using the Log-Rank test and Kaplan–Meier analysis. All data analyses were carried out using R software (version 4.2.2), and a two-sided *p*-value <0.05 was considered statistically significant.

## 3 Results

### 3.1 Clinical data of gastric cancer patients

The clinical information of the included patients with gastric cancer was present in Table 1. Among the 351 patients with advanced gastric cancer, 211 (60.11%) were PNI-positive, and 140 (39.89%) were PNI-negative. The average age of patients was 65.53 ± 10.19 years. The mean tumor thickness was 2.53 ± 1.06 cm, and the average HB level was 123.14 ± 27.82 g/L.

**Table 1:** Comparison of clinical and pathological data between the PNI-positive group and the PNI-negative group.

	PNI-negative Group (*n* = 140)	PNI-positive Group (*n* = 211)	t/z/χ^2^	*p*-value
Age (years)^a^	64 ± 10.74	66.55 ± 9.70	−2.310	0.021
Gender^b^			2.910	0.088
Male	44 (31.4%)	49 (23.2%)		
Female	96 (68.6%)	162 (76.8%)		
Endoscopic indicators				
Borrmann classification^b^			67.581	<0.001
I	41 (29.3%)	8 (3.8%)		
II	37 (26.4%)	30 (14.2%)		
III	31 (22.1%)	59 (28%)		
IV	31 (22.1%)	114 (54%)		
Tumor thickness (cm)^a^	2.32 ± 1.17	2.66 ± 0.96	−2.953	0.003
Histological differentiation^b^			49.797	<0.001
Highly differentiated	9 (6.4%)	0 (0%)		
Moderately differentiated	74 (52.9%)	51 (24.2%)		
Poorly differentiated	57 (40.7%)	160 (75.8%)		
Location of occurrence^b^			0.328	0.849
Gastric fundus	26 (18.6%)	41 (19.4%)		
Gastric body	42 (30%)	68 (32.2%)		
Gastric antrum	72 (51.4%)	102 (48.3%)		
Lauren classification^b^			38.658	<0.001
Intestinal type	68 (48.6%)	39 (18.5%)		
Mixed type	36 (25.7%)	65 (30.8%)		
Diffuse type	36 (25.7%)	107 (50.7%)		
Laboratory indicators				
CEA^b^ (ng/mL)			0.306	0.580
Normal reference range: 0–5				
≤5	111 (79.3%)	162 (76.8%)		
>5	29 (20.7%)	49 (23.2%)		
CA199^b^ (U/mL)			8.880	0.003
Normal reference range: 0–37				
≤37	128 (91.4%)	168 (79.6%)		
>37	12 (8.6%)	43 (20.4%)		
NLR^b^			11.400	0.001
≤2.75	92 (65.7%)	100 (47.4%)		
>2.75	48 (34.3%)	111 (52.6%)		
PLR^b^			6.000	0.014
≤177	99 (70.7%)	122 (57.8%)		
>177	41 (29.3%)	89 (42.2%)		
LMR^b^			9.175	0.002
≤3.44	43 (30.7%)	99 (46.9%)		
>3.44	97 (69.3%)	112 (53.1%)		
SIRI^b^			61.891	<0.001
≤1.31	123 (87.9%)	98 (46.4%)		
>1.31	17 (12.1%)	113 (53.6%)		
HB^a^ (g/L)				
Normal reference range: Male: 120–160 Female: 110–150	127.24 ± 24.86	120.42 ± 29.37	2.339	0.02
CT index				
EMVI^b^			56.350	<0.001
Negative	90 (64.3%)	51 (24.2%)		
Positive	50 (35.7%)	160 (75.8%)		
CT T Staging^b^			41.641	<0.001
ctT1-3	99 (75.7%)	75 (35.3%)		
ctT4	41 (29.3%)	136 (64.5%)		
CT enlarged lymph node ^b^			40.053	<0.001
Negative	79 (56.4%)	49 (23.2%)		
Positive	61 (43.6%)	162 (76.8%)		

a Represented independent sample *t*-test.

b Represented chi-square test, *p* < 0.05 indicated statistical significance.

In the PNI-positive group, 111 cases (52.6%) had an NLR > 2.75, while in the PNI-negative group, 48 cases (34.3%) had an NLR > 2.75. For PLR, 89 cases (42.2%) in the PNI-positive group had PLR > 177, compared to 41 cases (29.3%) in the PNI-negative group. LMR > 3.44 was observed in 112 cases (53.1%) in the PNI-positive group and 97 cases (69.3%) in the PNI-negative group. In the PNI-positive group, 113 cases (53.6%) had a SIRI >1.31, while in the PNI-negative group, 17 cases (12.1%) had a SIRI >1.31.

The probability of EMVI occurrence was 75.8% (160 cases) in the PNI-positive group and 35.7% (50 cases) in the PNI-negative group. Borrmann classification III-IV was observed in 235 cases (66.95%), with a PNI occurrence probability of 73.62%, while Borrmann classification I-II was observed in 116 cases (33.05%), with a PNI occurrence probability of 32.76%. ctT4 staging was present in 177 patients (50.43%) on CT, and lymph node enlargement was visible in 223 cases (63.53%). Tumor differentiation was moderate to high in 134 cases (38.18%) and low in 217 cases (61.82%). Elevated CEA and CA199 levels were observed in 22.22 and 15.67%, respectively. The majority (69.52%) exhibited a mixed or diffuse Lauren classification. Tumors were located in the gastric fundus (19.09%), body (31.34%), and antrum (49.57%).

### 3.2 Analysis of risk factors for PNI in advanced gastric cancer

The results of the univariate analysis revealed that age, EMVI, Borrmann classification, tumor thickness, CT T staging, CT enlarged lymph node, tissue differentiation, Lauren classification, CA199, NLR, PLR, LMR, SIRI, and HB were all significantly associated with the occurrence of PNI in advanced gastric cancer (*p* < 0.05), as shown in Table 1. Subsequently, the statistically significant indicators from the univariate analysis were included in a multivariate logistic regression model. The results indicated that EMVI, Borrmann classification, tumor thickness, and SIRI were independent risk factors for the occurrence of PNI in advanced gastric cancer (all *p* < 0.05), as presented in Table 2.

**Table 2:** Multivariate analysis of preoperative clinicopathological factors associated with PNI.

	*β*	Standard error	Wald	OR(95%CI)	*p*-value
**EMVI**					
Negative				Reference	
Positive	1.094	0.318	11.806	2.986 (1.600–5.573)	0.001
**Borrmann classification**					
I				Reference	
II	1.702	0.525	10.498	5.485 (1.959–15.359)	0.001
III	2.001	0.515	15.097	7.395 (2.695–20.288)	< 0.001
IV	2.208	0.516	18.316	9.101 (3.310–25.021)	< 0.001
Tumor thickness	0.295	0.130	5.134	1.343 (1.041–1.734)	0.023
**SIRI**					
≤1.31				Reference	
>1.31	1.882	0.323	34.019	6.564 (3.488–12.352)	< 0.001

### 3.3 Establishment and evaluation of the nomogram prediction model

**Figure 2:**
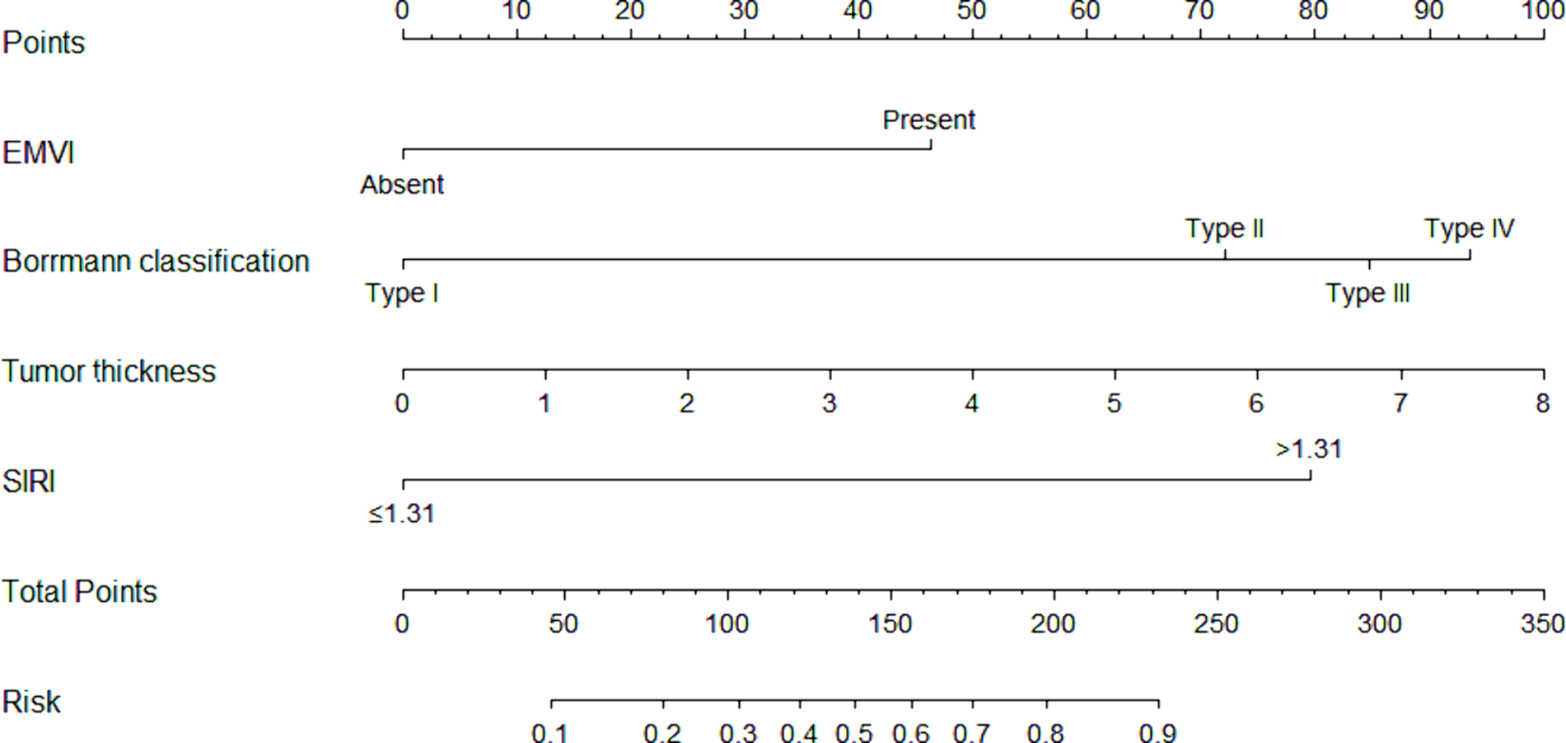
The nomogram prediction model. The nomogram prediction model included a total of four indicators. Preoperative endoscopic indicators consisted of Bormann classification and tumor thickness. CT indicators included EMVI. Laboratory indicators included SIRI. The scores for each variable were added together, and the corresponding numerical value of the total score represented the risk of PNI occurrence in gastric cancer.

Using R software, the predictive variables identified from the logistic regression analysis were incorporated into the nomogram prediction model. The outcome variable selected was the risk of PNI occurrence in advanced gastric cancer, resulting in the establishment of the preoperative nomogram prediction model for PNI in advanced gastric cancer (Figure 2).

The scoring system for the nomogram model was as follows: EMVI contributed 46 points, Borrmann classification I-IV contributed 0, 72, 85, and 94 points, respectively, and tumor thickness was graded from 0 to 8, corresponding to scores of 0, 12, 25, 38, 50, 62, 75, 88, and 100 points and SIRI >1.31 contributed 80 points. The score for each factor was obtained from the scale above the corresponding factor on the nomogram. The total score, obtained by summing the scores for each factor, corresponded to the probability of preoperatively predicting the occurrence of PNI in advanced gastric cancer.

**Figure 3:**
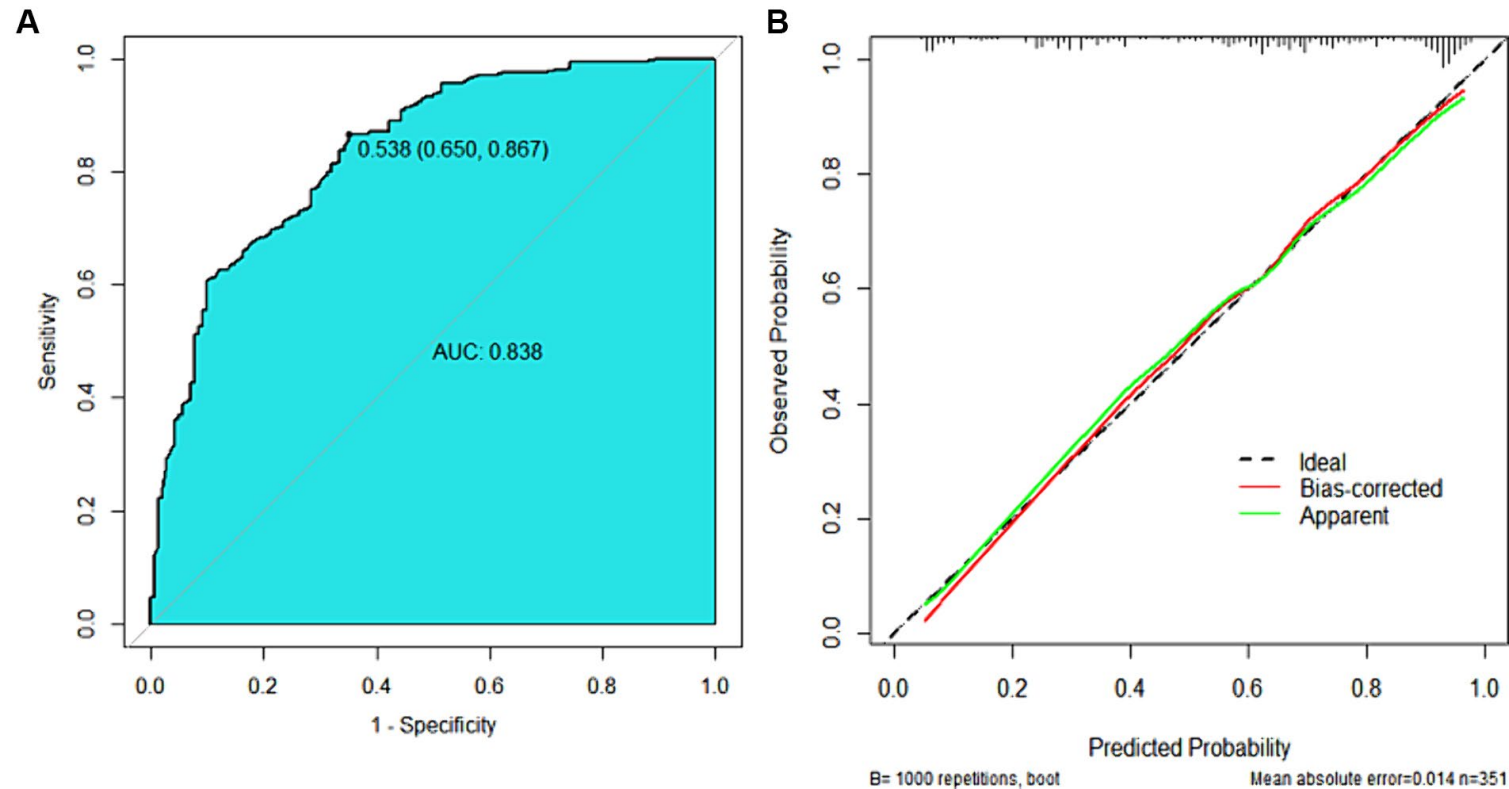
ROC curve and calibration curve of the nomogram prediction model. **(A)** ROC Curve, the AUC was 0.838 (0.795 ~ 0.880), the diagnostic threshold was 0.538, corresponding to a sensitivity of 86.7% and specificity of 65.0%. **(B)** Calibration Curve, the y-axis represented the actual probability of PNI, and the x-axis represented the predicted probability of PNI. The green line represented the fitted line for predicted probability corresponding to actual probability, the red line was the fitted line after removing errors from 1,000 internal validations, and the dashed line represented the calibration line of the ideal model.

**Figure 4:**
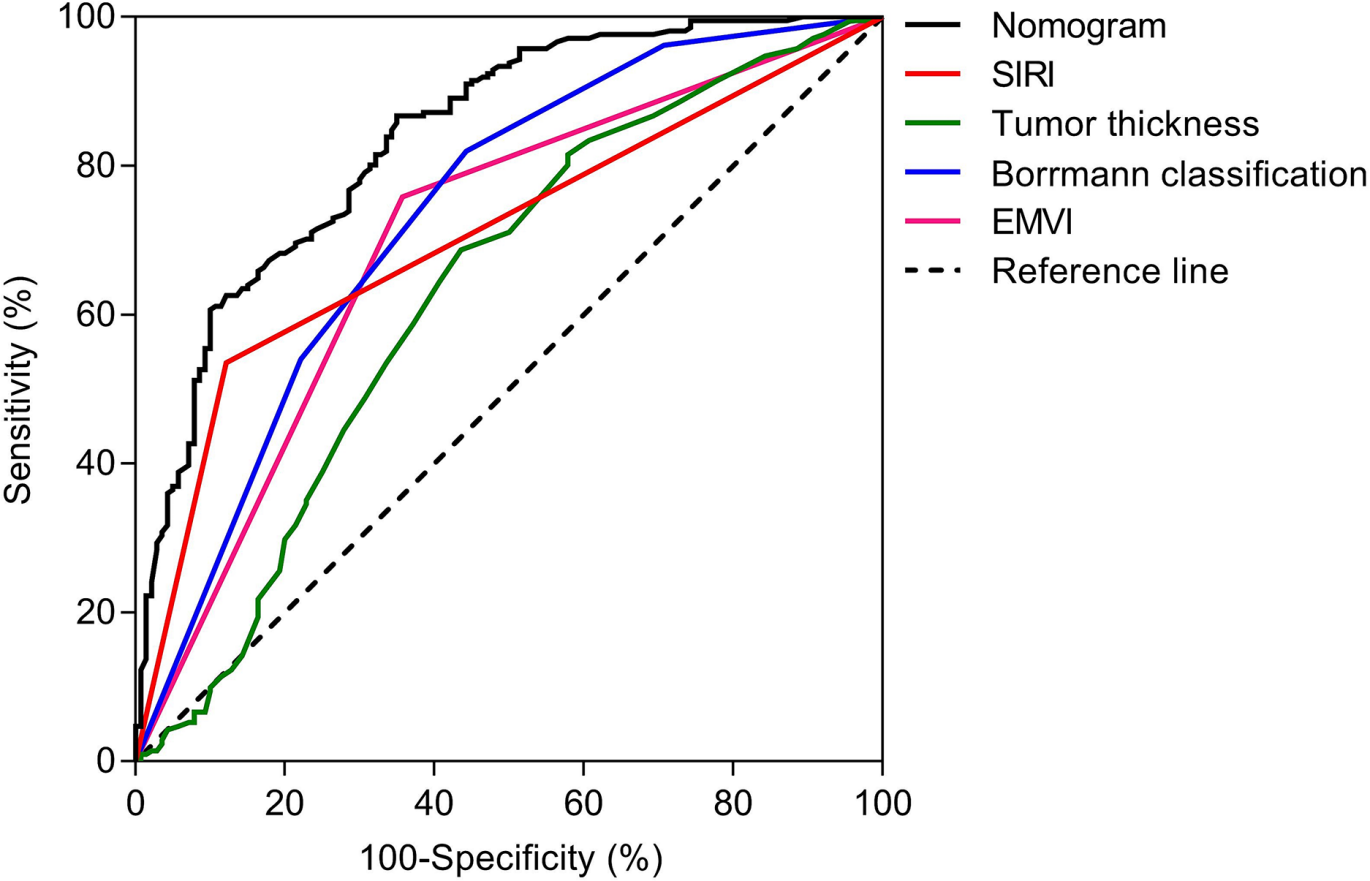
ROC analyses of nomogram and clinical indicators. The DeLong tests showed that the AUC value of nomogram was significantly higher than that of clinical indicators, and the differences were statistically significant (Z_EMVI_ = 6.554, Z_Borrmann classification_ = 4.751, Z_Tumor thickness_ = 6.033, Z_SIRI_ = 7.171, all *p* < 0.001).

**Figure 5:**
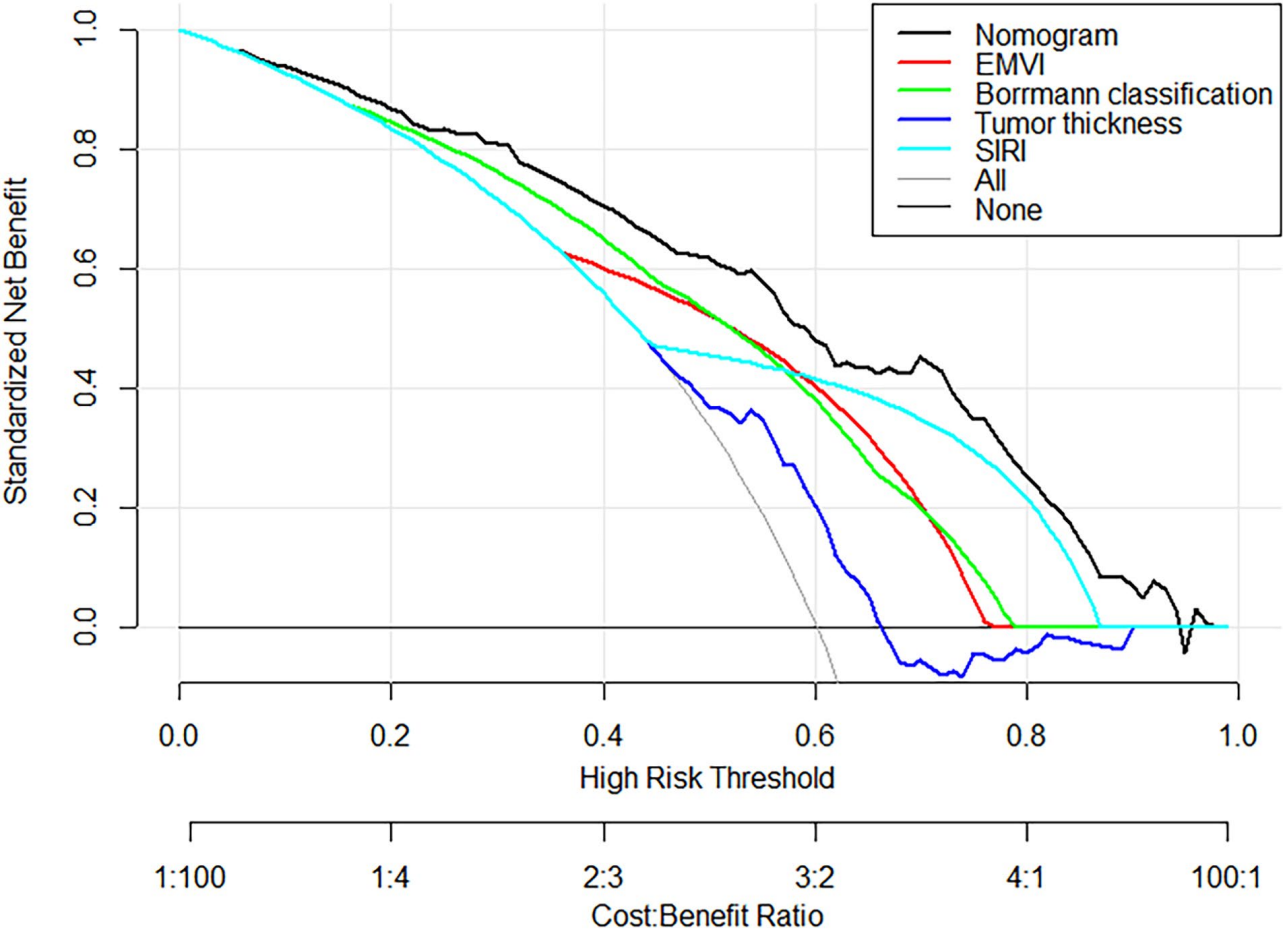
DCA Curves of the nomogram prediction model and other factors. DCA curves, the black curve represented the nomogram in the study, and other color curves were independent variables, such as EMVI, Borrmann classification, tumor thickness, and SIRI. Within the high-risk threshold range of 0.0 to 98.0, the net effect of the prediction model was higher than that of other factors.

The nomogram model offered good accuracy in estimating the risk of PNI with a C-index of 0.838 and a bootstrap-corrected C index of 0.814. ROC curve showed the AUC of the nomogram model was 0.838 (Figure 3A). DeLong tests revealed that the AUC of the nomogram model was significantly higher than that of clinical indicators, with statistically significant differences (Z_EMVI_ = 6.554, Z_Borrmann classification_ = 4.751, Z_Tumor thickness_ = 6.033, Z_SIRI_ = 7.171, all *p* < 0.001) (Figure 4; Table 3). The calibration curve demonstrated excellent consistency between the predicted and actual results (Figure 3B). The Hosmer-Lemeshow test for model fit indicated a good fit with *p* = 0.115. The DCA illustrated the model’s favorable clinical utility (Figure 5).

**Table 3:** ROC curves of nomogram prediction model and clinical indicators.

	AUC	95% CI	Sensitivity	Specificity	*p*-value
EMVI	0.701	0.643–0.758	0.758	0.643	<0.001
Borrmann classification	0.733	0.678–0.788	0.820	0.557	<0.001
Tumor thickness	0.630	0.568–0.691	0.687	0.564	<0.001
SIRI	0.707	0.653–0.761	0.536	0.879	<0.001
Nomogram	0.838	0.795–0.880	0.867	0.650	<0.001

### 3.4 Survival prediction

**Figure 6:**
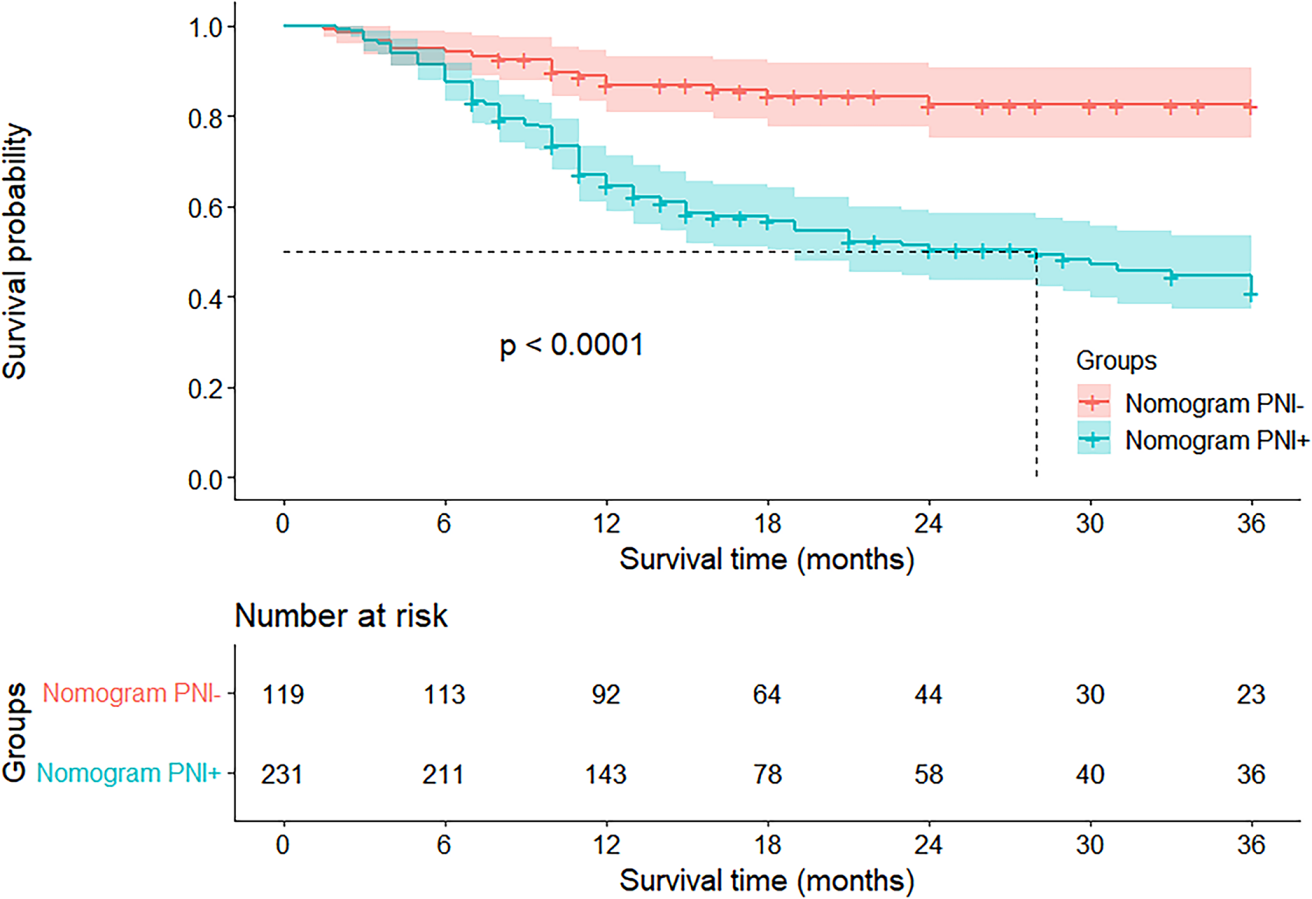
Kaplan–Meier survival curves according to nomogram-predicted PNI status. The Log-Rank test showed that the DFS of nomogram-predicted PNI-positive group was significantly lower than that of nomogram-predicted PNI-negative group, and the difference was statistically significant (*p* < 0.001).

In summary, we successfully followed up with 331 patients until September 31st, 2023. Over the follow-up period, 54 patients experienced disease progression, and 20 patients passed away. The mean DFS months for the nomogram-predicted PNI-positive group was 23.0, while for the nomogram-predicted PNI-negative group, it was 31.5 (*p* < 0.001). The Log-Rank test indicated a significantly lower DFS in the nomogram-predicted PNI-positive group compared to the nomogram-predicted PNI-negative group, with a statistically significant difference (*p* < 0.001) (Figure 6; Supplementary Table S2).

## 4 Discussion

This retrospective study introduced a preoperative nomogram aimed at personalized prediction of PNI in advanced gastric cancer prior to surgery, leveraging a combination of clinicopathological indicators and imaging parameters. The nomogram integrated endoscopic indicators such as tumor thickness and Borrmann classification, a imaging parameter like EMVI, and a laboratory indicator, namely the SIRI. This approach represented a straightforward, efficacious, and clinically relevant method for stratifying the risk of PNI and enhancing prognostic outcomes within the advanced gastric cancer population.

Gastric cancer is acknowledged as a highly heterogeneous tumor, with the incidence of PNI ranging from 6.8 to 75.6% (5, 8, 25, 26). The study encompassed a total of 351 gastric cancer patients, revealing a PNI incidence of 60.1% (211/351), consistent with existing literature reports.

The preoperative endoscopic indicators included in the nomogram model of this study were Borrmann classification and tumor thickness. Borrmann classification is a histological method. Díaz Del Arco et al. (27) stated that the Borrmann classification represented distinct biological morphology and reflected the aggressiveness of the tumor.

Ren et al. (11) discovered that Borrmann classification was an independent risk factor for PNI in advanced gastric cancer, with higher grades indicating a greater risk of PNI. This aligned with the findings of our study. Some studies reported that Lauren classification was an independent risk factor for PNI in advanced gastric cancer (10). However, these studies were based on pathological data from preoperative biopsy, which may differ from the postoperative pathological reality. In contrast, endoscopic Borrmann classification more intuitively reflected the morphological characteristics of the tumor and was more easily obtained than Lauren classification.

In our study, a significant correlation existed between tumor thickness and PNI in advanced gastric cancer. Tumor thickness reflected the growth cycle and depth of infiltration of the tumor. The deeper the infiltration, the higher the probability of contact with the abdominal nerve plexus, leading to a higher likelihood of PNI (28). In a recent study, it was suggested that the combination of tumor thickness and Borrmann classification may offer a more comprehensive depiction of tumor morphology and infiltration compared to relying solely on a single marker. This consideration arised from the recognition that tumors in gastric cancer rarely exhibited a spherical shape (29). The imaging parameter included in the nomogram model of this study was EMVI. Previous research indicated (23) a correlation between EMVI, lymph node metastasis, and poor postoperative prognosis in gastric cancer patients. PNI was also identified as an independent risk factor for adverse postoperative outcomes. In our study, results from univariate analysis showed a correlation between EMVI, CT enlarged lymph node, and PNI. However, multivariate analysis revealed that only EMVI was an independent risk factor influencing PNI in advanced gastric cancer.

EMVI refers to the direct invasion of the tumor into the venous vessels outside the intrinsic muscle layer of the gastrointestinal tract. It typically signifies deep tissue infiltration by the tumor and is considered a more serious manifestation of the disease. EMVI was widely applied in the diagnosis, treatment response assessment, and prognosis evaluation of various cancers, including colorectal and gastric cancers (30–33). A previous study (23) confirmed that EMVI was often associated with gastric cancer PNI and lymphovascular invasion (LVI), representing a mode of tumor spread along the neurovascular network.

Traditionally, EMVI can only be ascertained through postoperative histopathological examination, making it an impractical preoperative predictive parameter. However, Yang et al. (31) discovered that EMVI features can be obtained through non-invasive CT imaging. Therefore, in our present study, we utilized CT to assess the EMVI status, providing a more convenient and comprehensive observation angle compared to endoscopic biopsy.

This study represented the first discovery of a significant association between SIRI, a novel inflammation biomarker, and PNI in advanced gastric cancer. SIRI was calculated based on the counts of neutrophils, monocytes, and lymphocytes in peripheral blood, accurately reflecting the tumor’s inflammatory status. Notably, it demonstrated a superior ability to assess the prognosis of gastric cancer patients compared to other traditional inflammatory indicators (34).

Previous research confirmed (14–16, 35) the close correlation between inflammation and the progression and metastasis of tumors. Tumor cells possess the capability to engage with the tumor microenvironment (TME) through the secretion of diverse inflammatory factors and the production of specific chemokines and adhesion factors. These actions facilitate the migration towards peripheral nerves, thereby promoting neural invasion. Additionally, these cytokines can activate certain signaling pathways, accelerating the invasion of tumor cells into neural tissue (4, 7). Hence, inflammation may facilitate the occurrence of PNI in gastric cancer. The inflammatory status of the tumor can be reflected through inflammatory markers in peripheral blood (35), prompting a comprehensive analysis in this study of the correlation between peripheral blood inflammatory markers and PNI.

Results from univariate analysis indicated that NLR, PLR, LMR, and SIRI were associated with PNI. However, multivariate analysis revealed that only SIRI was a risk factor for PNI in advanced gastric cancer. Consequently, patients with high SIRI levels in this study were more prone to experiencing PNI, and the predictive performance of SIRI for PNI surpassed that of other traditional inflammatory markers.

The nomogram model in this study demonstrated a favorable identification effect for both PNI-positive and PNI-negative cases, boasting an AUC value of 0.838. The calibration curve and decision curve analyses further affirmed the nomogram model’s excellent discriminatory ability and clinical utility. Several other prediction models were developed for predicting PNI in advanced gastric cancer, including one by Liu et al. (36). However, their clinical prediction model lacked inflammation-related parameters. Moreover, some of the included indicators, Lauren classification (diffuse type) and cT4 stage did not meet statistical screening criteria in the multivariate analysis of that research.

Li et al. (29) developed a PNI prediction model for advanced gastric cancer based on preoperative imaging parameters. However, their study heavily relied on imaging parameters while including minimal clinical indicators, and the interpretation of parameters may be biased due to variations in image quality across different imaging devices.

A recent study (28) found that a radiomics model based on imaging data can successfully predict the occurrence of PNI in advanced gastric cancer. Nonetheless, this model encountered challenges, including limited generalization and difficulties in feature extraction. In comparison to previous studies, the nomogram model in this research employed a diverse array of examination techniques, including endoscopy, CT scans, tumor marker detection, and inflammation index assessment. The primary emphasis was placed on exploring the connection between preoperative clinical factors and certain imaging indicators in gastric cancer patients and PNI. The encompassed clinical indicators were notably comprehensive and objective, utilizing a data acquisition method that was thorough, convenient, and reliable. This approach was conducive to the creation of a genuinely reliable prediction model before treatment, facilitating clinicians in making accurate and informed clinical decisions.

This study delved into the prognostic implications of the preoperative nomogram model, revealing a significantly lower DFS in the nomogram-predicted PNI-positive group compared to the nomogram-predicted PNI-negative group, as determined by the Log-Rank test and Kaplan–Meier analysis. Previous research consistently affirmed PNI as a prognostic factor for gastric cancer patients, with postoperative overall survival (OS) and DFS notably reduced in patients with PNI compared to those without PNI (8, 12).

The underlying mechanism through which PNI influenced the prognosis of gastric cancer patients involved a complex interplay between cancer cells and peripheral nerves, forming a detrimental cycle. Cancer cells induced nerve damage, and in turn, damaged nerves promoted cancer cell spread by releasing various cytokines and chemokines. This dynamic process accelerated disease progression, ultimately leading to lower postoperative survival rates (37–39).

Recent findings suggested that patients with PNI who undergone adjuvant chemotherapy experience improved postoperative OS and DFS (40). Hence, the nomogram model presented here not only effectively predicted preoperative PNI status but also contributed to postoperative risk stratification. This held significant implications for developing personalized treatment plans and enhancing the overall prognosis of gastric cancer patients.

This study had several limitations. Firstly, it was a single-center retrospective study with a small sample size, which might introduce selection bias. The predictive model underwent internal cross-validation but lacked external validation, highlighting the need for further validation using multi-center large datasets. Secondly, although the predictive model in this study incorporated multiple clinical and imaging indicators, it remained incomplete. Future efforts should focus on the inclusion of quantitative parameters derived from clinical and imaging data to enhance the preoperative prediction of PNI in advanced gastric cancer.

## 5 Conclusion

In this study, we successfully constructed a nomogram model based on preoperative endoscopic, imaging, and laboratory indicators of patients. This model was utilized for predicting PNI in advanced-stage gastric cancer. It represented an efficient, convenient, and highly operational approach. The model aided in guiding postoperative treatment strategies for gastric cancer patients, thereby enhancing the prognosis of advanced-stage gastric cancer, improving patient survival time, and enhancing overall quality of life.

## Data availability statement

The original contributions presented in the study are included in the article/Supplementary material, further inquiries can be directed to the corresponding author.

## Author contributions

RC: Conceptualization, Data curation, Formal analysis, Funding acquisition, Investigation, Methodology, Software, Validation, Writing – original draft, Writing – review & editing. RX: Conceptualization, Data curation, Formal analysis, Visualization, Writing – original draft. JM: Software, Validation, Writing – original draft. ZZ: Formal analysis, Funding acquisition, Investigation, Supervision, Validation, Writing – review & editing.
