# Supplementary material for: Construction of a preoperative nomogram model for predicting perineural invasion in advanced gastric cancer
Source: Front Med (Lausanne). 2024 Jun 7;11:1344982. doi: 10.3389/fmed.2024.1344982 (PMC11190154; doi:10.3389/fmed.2024.1344982)
Supplement: Supplementary file 1 [file Data_Sheet_1.DOC]

**Table S1: ROC Curves of the Inflammation Parameters Predicting PNI in Advanced Gastric Cancer**

| Inflammation parameters | AUC (95% CI) | Cut-off value | Sensitivity | Specificity |
| --- | --- | --- | --- | --- |
| NLR | 0.602 (0.549-0.654) | 2.75 | 0.540 | 0.671 |
| PLR | 0.576 (0.522-0.628) | 177 | 0.422 | 0.721 |
| LMR | 0.558 (0.505-0.611) | 3.44 | 0.469 | 0.693 |
| SIRI | 0.586 (0.532-0.638) | 1.31 | 0.427 | 0.771 |

**Table S2: Comparison of Disease-Free Survival Probability According to Nomogram-predicted PNI Status**

| Groups | Mean DFS,  Months  (95% CI) | 1. year DFS   rate(%) | 1. year DFS   rate(%) | 1. year DFS   rate(%) | Log-rank | *P* |
| --- | --- | --- | --- | --- | --- | --- |
|  |  |  |  |  | 30.493 | <0.001 |
| Nomogram-predicted PNI-negative | 31.5  (29.6, 33.4) | 88.8 | 84.3 | 82.4 |  |  |
| Nomogram-predicted PNI-positive | 23.0  (21.2, 24.9) | 67.0 | 51.4 | 40.9 |  |  |
